# The patients’ experience of neuroimaging of primary brain tumors: a cross-sectional survey study

**DOI:** 10.1007/s11060-023-04290-x

**Published:** 2023-03-28

**Authors:** Ivar J. H. G. Wamelink, Hugo L. Hempel, Elsmarieke van de Giessen, Mark H. M. Vries, Philip De Witt Hamer, Frederik Barkhof, Vera C. Keil

**Affiliations:** 1 Radiology & Nuclear Medicine Department, Amsterdam UMC Location Vrije Universiteit Amsterdam, De Boelelaan 1117, 1081 HV Amsterdam, The Netherlands; 2 Cancer Center Amsterdam, Brain Tumor Center Amsterdam, Amsterdam, The Netherlands; 3 Amsterdam Neuroscience, Brain Imaging, De Boelelaan 1117, Amsterdam, The Netherlands; 4 Amsterdam UMC Location Vrije Universiteit Amsterdam, De Boelelaan 1117, Amsterdam, The Netherlands; 5 Queen Square Institute of Neurology and Centre for Medical Image Computing, University College London, London, UK

**Keywords:** Primary brain neoplasms, Magnetic resonance imaging, Survey, Gadolinium

## Abstract

**Purpose:**

To gain insight into how patients with primary brain tumors experience MRI, follow-up protocols, and gadolinium-based contrast agent (GBCA) use.

**Methods:**

Primary brain tumor patients answered a survey after their MRI exam. Questions were analyzed to determine trends in patients’ experience regarding the scan itself, follow-up frequency, and the use of GBCAs. Subgroup analysis was performed on sex, lesion grade, age, and the number of scans. Subgroup comparison was made using the Pearson chi-square test and the Mann–Whitney U-test for categorical and ordinal questions, respectively.

**Results:**

Of the 100 patients, 93 had a histopathologically confirmed diagnosis, and seven were considered to have a slow-growing low-grade tumor after multidisciplinary assessment and follow-up. 61/100 patients were male, with a mean age ± standard deviation of 44 ± 14 years and 46 ± 13 years for the females. Fifty-nine patients had low-grade tumors. Patients consistently underestimated the number of their previous scans. 92% of primary brain tumor patients did not experience the MRI as bothering and 78% would not change the number of follow-up MRIs. 63% of the patients would prefer GBCA-free MRI scans if diagnostically equally accurate. Women found the MRI and receiving intravenous cannulas significantly more uncomfortable than men (*p* = 0.003). Age, diagnosis, and the number of previous scans had no relevant impact on the patient experience.

**Conclusion:**

Patients with primary brain tumors experienced current neuro-oncological MRI practice as positive. Especially women would, however, prefer GBCA-free imaging if diagnostically equally accurate. Patient knowledge of GBCAs was limited, indicating improvable patient information.

**Supplementary Information:**

The online version contains supplementary material available at 10.1007/s11060-023-04290-x.

## Introduction

Patients with primary brain tumors, especially with gliomas, usually receive multiple MRI scans per year as standard care. Patients with a slow-growing low-grade glioma (LGG) may undergo dozens of MRI scans due to their chronic condition. Research endeavors towards faster and more informative MRI protocols are ongoing [1–4], but glioma MRI protocols remain lengthy and include gadolinium-based contrast agents (GBCA). The patient opinion on radiological care is largely unknown, despite the vulnerability of glioma patients and the relevant implications for patients and physicians.

MRI is a crucial pillar of therapy planning and response evaluation in neuro-oncology [5]. Brain tumor MRI protocols tend to adhere to consensus recommendations [6]. Most guidelines include an initial follow-up interval between three to six months after the completion of therapy, depending on the tumor histology. The scanning interval should be decreased to four to eight weeks in case of possible disease progression [7]. However, patients with brain tumors undergo particularly long and frequent MRI scans, while many low-grade brain tumors remain stable for long periods [8–10]. Furthermore, the benefit of fixed interval imaging remains unclear [11, 12].

Contrast-enhanced T1 weighted imaging (CET1w), often including contrast-enhanced dynamic susceptibility contrast perfusion imaging (DSC), is considered invaluable to the toolbox of neuroradiologists and is standard of care during the follow-up of brain tumors. However, research has shown long-term GBCA deposition, and current patient claims of GBCA-induced side effects are under investigation [13, 14]. Therefore, American and European pharmaco-safety agencies urge clinicians only to use GBCA when strictly necessary, but risk–benefit analyses for GBCA are awaited [15–17]. Many lesions never enhance or enhance without being high-grade brain tumors.

Against this backdrop, it becomes clear why neuroradiological research focuses on strategies to optimize imaging intervals. It also explores using advanced MRI sequences and artificial intelligence to shorten scan protocols and gain deeper insight into tumor biology [18–24]. This includes imaging without or with reduced GBCA, particularly for the low-grade tumor follow-up in the pediatric population [25–27].

The patient opinion on radiological care in brain tumor management is mainly unknown despite a general acknowledgement of the value of patient-centered research and shared decision-making [28, 29]. This includes patient opinions on GBCA use.

There is a knowledge gap regarding the opinion of the patient on neuro-oncological MRI and research developments in particular, which also has a negative impact on the planning of future MRI research lines.

To gain more insight into the patient perspective on neuro-oncological MRI, its follow-up, and the use of GBCAs and to draw conclusions on the patient-perceived urgency of current research lines, we performed a cross-sectional survey on patients with primary brain tumors.

## Methods

### Study design and participants

The local ethics committee approved the study. A questionnaire was designed in collaboration with our patient-reported experience measures department. One hundred patients were estimated as a sufficient sample size following the COSMIN study design checklist for patient-reported outcome measurement instruments [30].

Questionnaire targets were adult primary intra-axial brain tumor patients with at least 1 year of known diagnosis who had regular follow-up at our institution. The diagnosis had been histopathologically confirmed or was based on radiological phenotype and multidisciplinary consensus (“scan and wait”). Impairments due to tumor therapy were not an exclusion criterion, nor a selection criterion. All patients that had a neuro-oncological MRI scan and met all the inclusion-criteria were consecutively approached before their clinical MRI scan between 01-09-2021 and 04-08-2022. Patients needed to give informed consent before the clinically scheduled regular MRI scan and were interviewed directly after the scan. Patients with acute impairment, e.g. due to recent brain surgery (early postoperative MRI), were excluded from recruitment, as were patients under legal guardianship. The questionnaire was conducted on Dutch-speaking patients only. Participation was voluntary, and patients did not receive any compensation.

Additional information on sex, age at study participation, tumor type and therapy course were added based on medical records. Grade 4 and 3 lesions were classified as high-grade gliomas (HGG), as most lesions were glioma-type. All others were in the LGG category. The WHO classification at the time of surgery defined the diagnosis. The number of previous MRI scans was registered from electronic hospital notes.

### Questionnaire

Originally, the questionnaire contained ten questions plus one open comment space. It was extended by one additional question (question 11) during the course of the study in order to gain additional information on patient knowledge of GBCA.

First, the patient was asked to estimate their total number of tumor-related brain MRIs received until present. This was to evaluate if patients realistically assess the burden of MRI during the disease. The other questions required single-choice categorical and ordinal tick-box answers. Eight out of eleven questions allowed multiple answer options.

Questions 2 and 10 were general questions on the burden of undergoing radiological follow-up as a patient with a primary brain tumor. Questions 3–6 covered the dimension of burden due to GBCA injection. Questions 7–9 covered the burden of the MRI scan procedure beyond GBCA injection. An open comment section ended the questionnaire. These comments were categorized into four groups: general burden of MRI, attitude towards GBCA injection, the burden due to follow-up/scan interval, and others.

The English version of the PENGUIN questionnaire can be found as Online Resource 1.

### Analysis

Data was analyzed with descriptive statistics. Subgroup analyses were performed for sex, age, tumor grade, and the number of follow-up scans. Question 10 was transformed to a binary metric during the analysis, as patients were allowed to give multiple answers. If patients left all the boxes empty, they were, after confirmation by the patient, categorized as not having any stress. The subgroup comparison involved parametric testing with the Pearson chi-square test and the Mann–Whitney U-test for categorical and ordinal questions, respectively. We also performed the Pearson correlation test. Bonferroni correction was performed for multiplicity. The significance level was set by dividing the significance threshold (0.05) by the number of subgroups (4) at *p* < 0.01.

## Results

### Patient demographics

Of the one hundred filled-in questionnaires, 61 were from male patients (mean age ± standard deviation (SD): 44 ± 14 years) and 39 from female patients (mean age ± SD: 46 ± 13 years, Table 1). The age difference was insignificant between male and female patients (*p* = 0.4). Ninety-three patients had a histopathologically confirmed diagnosis. The other seven patients were considered to have a slow-growing low-grade lesion based on their radiophenotype and growth rates. In total, 41 patients had a HGG. Online Resource 2 shows the number of patients for each tumor entity.

**Table 1:** Patient subgroups split by sex and age for lesion subtypes and number of follow-up scans

Age (in yrs.)^a^	# of Patients	Male (n = 61)			Female (n = 39)		
		< 35	35–55	55 +	< 35	35–55	55 +
High-grade	41	6	13	10	3	3	6
Low-grade	59	15	13	4	8	13	6
< 10 scans	33	8	8	3	4	5	5
10–20 scans	39	10	9	8	2	6	4
20–30 scans	18	2	7	3	3	3	0
> 30 scans	10	1	2	0	2	2	3

^a^Thirty-two patients were younger than 35 while 26 patients were older than 55. Forty-two patients were between the ages of 35 and 55

### General burden of MRI

**Fig. 1:**
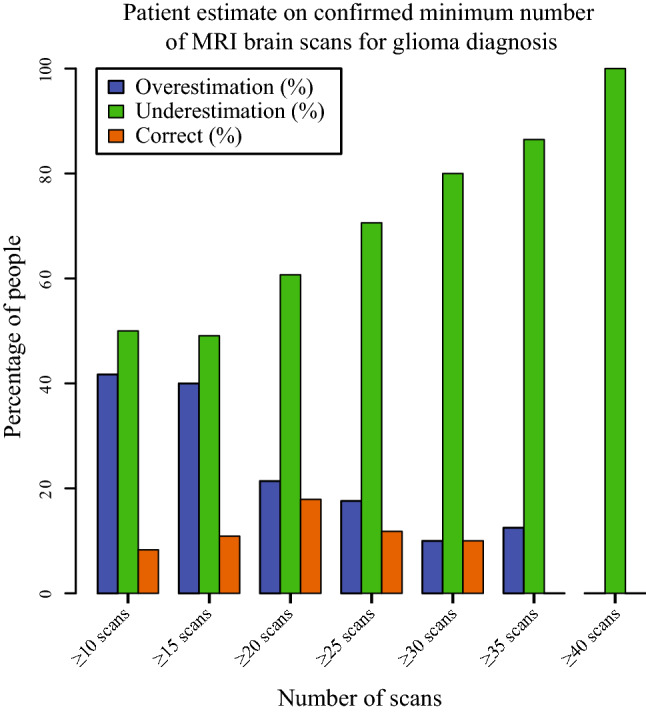
Percentage of patients overestimating, underestimating, or guessing correctly the number of MRI for glioma they had undergone until questionnaire session (y-axis) as sorted by the number of MRI they had truly undergone (x-axis). Underestimation of scan burden increases with the number of MRI undergone

Patients systematically underestimated the number of scans they had undergone (49 underestimations vs 39 overestimations). An increase in the number of follow-up scans was associated with an increased underestimation of MRI burden (Fig. 1). If overestimation occurred, it was more marked than in cases of underestimation: 5 + 6.57 scans overestimated compared to 3 + 4 scans (median + interquartile range) underestimated, respectively. There was no significant difference in over- or underestimation between men and women (*p* = 0.83).

**Fig. 2:**
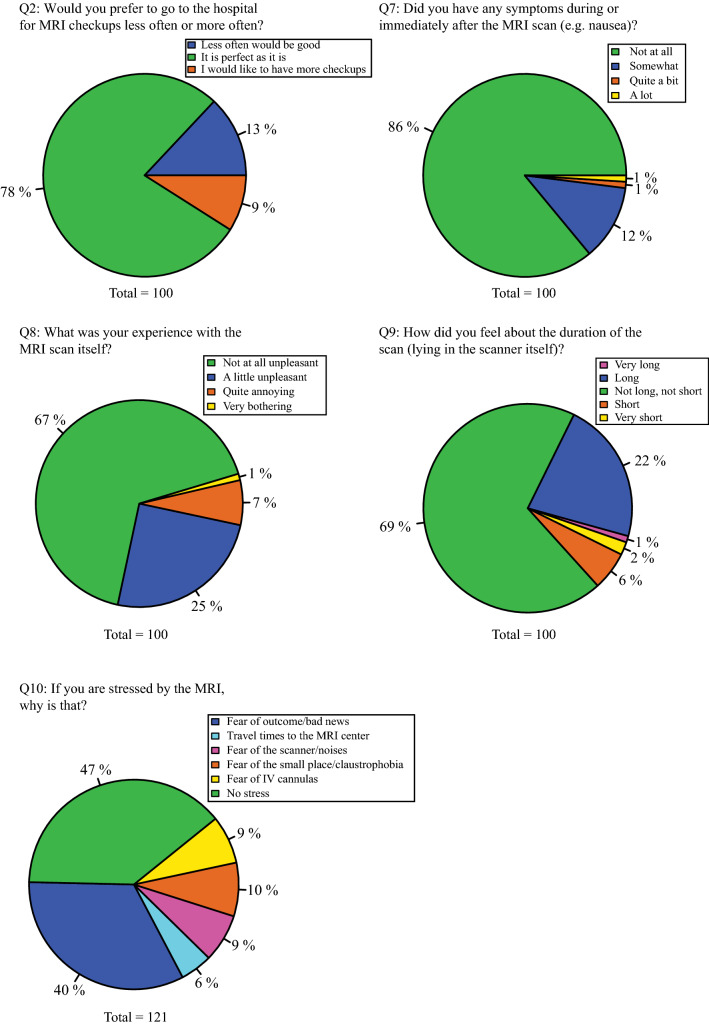
Questions 2 and 7–10 measured the dimensions of the general burden of the MRI. Patients were allowed to give multiple answers to question 10 if they experienced several types of stress

The general trend was that patients did not consider MRI scans burdensome, as shown in Fig. 2. It appeared that older patients experienced lying in the MRI as longer than younger patients, albeit not significantly (Q9, Online Resource 3). The most frequent stress factor was fear of outcome/bad news (Fig. 2). Five patients found the noise annoying or suggested extra hearing protection beyond the existing double layer.

Women reported more symptoms during or directly after the MRI (Q7) and found it more annoying than males (Q8). Women also experienced more stress from fearing bad news (Q10.1) and the travel times to the MRI unit (Q10.2) and experienced more stress in general (Q10.6). Patients with less than ten previous MRIs were significantly more dissatisfied regarding the scan follow-up interval than patients with 30 or more scans. The same group (< 10 scans) showed significantly less fear towards receiving an intravenous (IV) cannula. Finally, LGG patients experienced more claustrophobia than HGG (Online Resource 3).

### Attitude towards GBCA injection

**Fig. 3:**
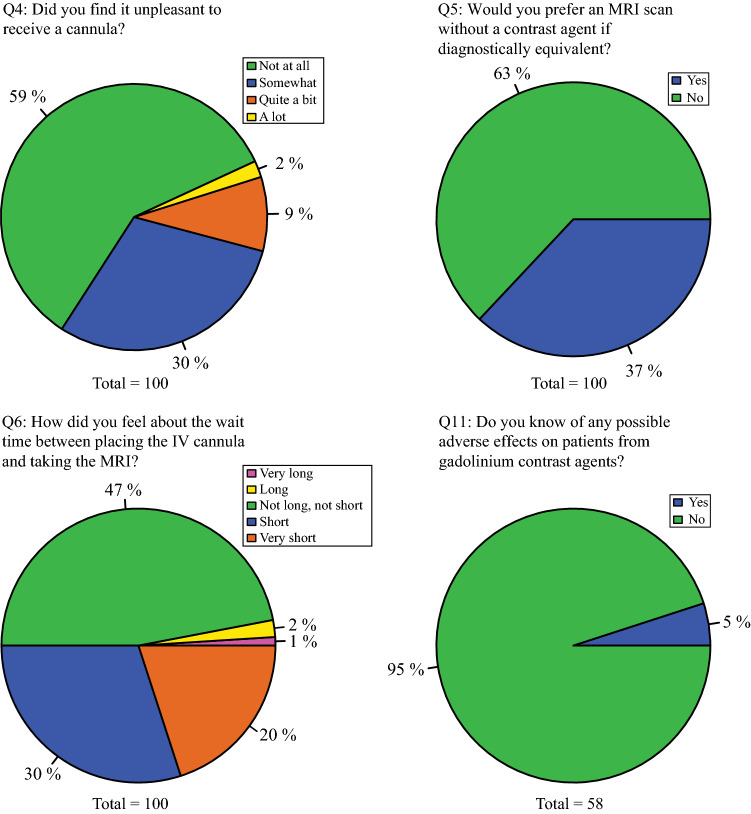
Questions 4–6 and 11 explore the dimensions of attitude towards GBCA injection

Most patients did not experience GBCA injections as burdensome (Fig. 3). However, 40% described at least some irritation when receiving IV administration. 63% preferred an MRI scan without GBCA if considered diagnostically equivalent. Fifty-eight patients answered the additional question about possible adverse effects from GBCA, but only three patients were aware of these. Nearly all patients found the wait between placing the cannula and taking the MRI perfect or short. Several patients wrote that they found cannulas annoying and painful and would prefer no cannula if diagnostic performance was maintained. One patient also mentioned that patients should receive more information regarding the use of GBCA agents.

Female patients found cannula placement significantly more unpleasant than males (Q4). There were no significant differences between males and females regarding preferences for MRI options without GBCA (Q5).

## Discussion

Patients with primary brain tumors expressed a generally positive attitude towards the current neuro-oncological MRI follow-up scheme in this monocentric survey at a tertiary academic center. However, GBCA-free MRI protocols would be preferred, provided their diagnostic non-inferiority. Importantly, patient knowledge about any potential adverse effects of GBCA was rare, and we identified women as less satisfied. At the same time, age, diagnosis and number of previous scans had no impact on satisfaction.

Patients underestimated the number of scans they had undergone, with underestimation being positively correlated with the number of previous scans. Scan burden underestimation can be explained by ‘positivity bias in memory’—a phenomenon describing a person’s inclination to remember pleasant events more vividly and favorably than unpleasant ones [31, 32]. Patients with 30 scans or more were significantly more satisfied with the number of scan follow-ups than patients with ten scans or fewer. We hypothesize that patients with more scans are more likely to think they are in a stable phase of their disease than patients who only recently got diagnosed. There is a tendency in the medical community to reduce both MRI frequency and protocol duration, scan time. Arguments are costs, waiting lists, and the assumption that patients find the MRI uncomfortable and have difficulty complying [33–35]. However, our results showed that most patients did not experience MRI as burdensome and that the follow-up intervals are perceived as appropriate—even by frequently scanned glioblastoma patients. The debate about whether scan frequency and protocol duration need to be reduced should include the patients, as they might oppose longer control intervals. On the other hand, data implies that most patients will tolerate moderate scan duration extension for imaging research.

However, certain patients experienced at least moderate discomfort during the MRI scan. It is worthwhile to study this group in more detail.

Our most remarkable and also most consistent finding is the role of sex in the perception of MRI. Overall, women found the MRI procedure more uncomfortable than men, which was characterized by experiencing the MRI procedure as more unpleasant, more often being afraid of bad news, and having a tendency to be more stressed about the travel times. These findings align with literature suggesting that women experience more stress and anxiety also when confronted with a brain tumor diagnosis [36, 37]. Women also found receiving a cannula more unpleasant. Research has reported that sex is a risk factor for difficult venous access and that catheter insertion in women is more difficult, explaining the difference in comfort [38]. We conclude that sex, and most likely gender, is not sufficiently reflected in the current MRI workflow of brain tumor patients despite indicators for relevant differences between male and female perception. According to our results, women will benefit from shorter MRI protocols–and should innovation permit it–even GBCA-free ones. The discussion between patient welfare and patient clinical needs should therefore be carefully balanced.

The age, number of previous scans, and diagnosis had surprisingly little impact on patients’ MRI perception. Increasing age is a known stress factor for patients and MRI technicians [39]. In our study, we could only confirm a tendency below the significance threshold regarding age: older patients tended to experience the MRI scan as longer and less comfortable than younger patients. This is relevant as brain tumor MRI protocols are particularly lengthy.

Patients with ten or fewer scans showed less fear of receiving a cannula than patients with 30 scans or more, at a significant level before and a possibly still relevant level after Bonferroni correction. While the probability of a negative experience with cannulas increases with the growing number of scans, this contrasts with what would be expected as dictated by exposure therapy. With exposure therapy, frequent engagement with anxiety-provoking stimuli, such as cannulas, can reduce and disconfirm a person’s fearful projections towards the respective stimulus [40, 41].

Patients with a low-grade lesion experienced more claustrophobia than patients with a high-grade lesion. Patients in the low-grade group had a mean age of 41 years, while patients in the high-grade group had a mean age of 49, as expected. Even though the age differences between the two groups were normally distributed, the age difference could explain this finding as younger patients tend to be more stressed [42]. As younger patients with low-grade lesions will likely receive more follow-ups during their life time, any scientific innovation towards shorter protocols will be particularly in their favor. Clinically, the time between scans, the number of included sequences, and the decision of administering contrast is generally based on the lesion type. However, our results show that tumor type, a reflection of disease severity, does not play a relevant role in the perception of MRI. While our research did not focus on the patient-disease relationship, there may be a link between the patients’ tolerance for number of follow-up scans and scan duration and the type of disease.

An estimated ~ 40% of all MRI scans in neuroradiology are GBCA-dependent [43]. Therefore, patient opinion on gadolinium should be considered with the aim of shared decision-making [44]. Our results show that most patients would opt for an MRI scan without GBCAs if considered diagnostically non-inferior, supporting research in that direction. However, patients showed a profound lack of knowledge of GBCAs, including insufficient knowledge regarding possible adverse effects despite being a frequently prescribed diagnostic agent. Patients seemed to be poorly informed and could thus not make optimal decisions about their welfare. At this point, it must be understood that patients in the Netherlands will usually never meet with a radiologist, nor is written informed consent for MRI examinations with GBCA mandatory. Potential contraindications for MRI are ruled out by the clinician ordering the MRI scan. A detailed procedure description for the patient is usually not part of this conversation. Especially considering that pharmaco-safety agencies urge clinicians to reduce the use of gadolinium in the clinical workflow of glioma patients [16, 45], patients should be well-informed about the added value of GBCAs and their possible harm to the human body [13]. Beyond considerate use of GBCA and conciseness of scan protocols, there are other factors which may be relevant to increase patient comfort such as an acoustic optimization of sequences, as was confirmed by the comments of five of our participants [46].

There are several limitations to this study. First, this is a monocentric study with Dutch patients only, with consequences for data interpretation. The sample was, however, representative of the disease's general prevalence regarding diagnosis, age, and sex distribution [47]. Second, some of our patients have outdated diagnosis without an IDH classification which may result in high grade glioma patients being considered low grade glioma. Patients for whom the questionnaire was too much of a burden were excluded, as were non-Dutch-speaking patients due to the language barrier. This biases the study, potentially underestimating the MRI burden by excluding the sickest patients and patients with a different cultural background. Further, the reference number of MRI scans derived from hospital records is a minimum estimate. Patients could also have been scanned elsewhere. However, patients in the Netherlands usually adhere to one clinic only for treatment and follow-up—making a relevant deviation in the correct total number of scans unlikely.

In summary, this study finds that patients with primary brain tumors generally have positive experiences with neuro-oncological MRI. Especially women, however, would support endeavors towards GBCA-free MRI diagnostics and shorter protocols. Approaches to reduce imaging frequency are neither a patient priority, nor preference. A lack of knowledge on GBCA indicates that shared decision-making remains an unreached goal in glioma imaging.

## Supplementary Information

Below is the link to the electronic supplementary material.

Supplementary file1 (PDF 200 KB)

Supplementary file2 (PDF 153 KB)

Supplementary file3 (PDF 134 KB)

## Data Availability

The datasets generated during and/or analyzed during the current study are available from the corresponding author on reasonable request.

## Abbreviations

CET1w: Contrast-enhanced T1 weighted imaging
DSC: Dynamic susceptibility contrast
EORTC: European organization for research and treatment of cancer
GBCA: Gadolinium-based contrast agent
HGG: High-grade glioma
IV: Intravenous
LGG: Low-grade glioma
SD: Standard deviation
WHO: World health organization
