# Supplementary material for: The patients’ experience of neuroimaging of primary brain tumors: a cross-sectional survey study
Source: J Neurooncol. 2023 Mar 28;162(2):307–15. doi: 10.1007/s11060-023-04290-x (PMC10167184; doi:10.1007/s11060-023-04290-x)
Supplement: Supplementary file 1 — Supplementary file1 (PDF 200 KB) [file 11060_2023_4290_MOESM1_ESM.pdf]

English version:

Questionnaire PENGUIN study

1. How many MRI brain scans have you had: ______________ times.

2. Would you prefer to go to the hospital for MRI checkups less often or more often?
- **Less often would be good**
- **It is perfect as it is**
- **I would like to have more checkups**

3. Did you receive a contrast injection?
- **Yes**
- **No**

4. Did you find it unpleasant to receive a cannula?
- **Not at all**
- **Somewhat**
- **Quite a bit**
- **A lot**

5. Would you prefer to have an MRI scan without a contrast agent, if diagnostically equivalent?
- **Yes**
- **No**

6. How did you feel about the wait time between placing the IV cannula and taking the MRI?
- **Very long**
- **Long**
- **Not long, not short**
- **Short**
- **Very short**

7. Did you have any symptoms during or immediately after the MRI scan (e.g., nausea)?
- **Not at all**
- **Somewhat**
- **Quite a bit**
- **A lot**

8. What was your experience with the MRI scan itself?
- **Not at all unpleasant**
- **A little unpleasant**
- **Quite annoying**
- **Very bothering**

9. How did you feel about the duration of the MRI scan (lying in the scanner itself)?

- **Very long**
- **Long**
- **Not long, not short**
- **Short**
- **Very short**

10. If you are stressed by the MRI, why is that?

- **Fear of outcome/bad news**
- **Travel times to the MRI center**
- **Fear of the scan/machine itself/noises**
- **Fear of the small space/claustrophobia**
- **Fear of IV cannulas**

11. Do you know of any possible adverse effects on patients from gadolinium contrast agents?

□ **Yes**
□ **No**

12. Here is space for your own comments:

____

____

____

____

<u>Dutch version:</u>

Vragenlijst PENGUIN studie

1. **Vooraf: Hoeveel MRI hersenscans heeft u ruim gehad: ______________** keer

2. **Zou u liever minder vaak of vaker naar het ziekenhuis gaan voor MRI-controles?**
   - **Minder vaak zou goed zijn**
   - **Het is perfect zo**
   - **Ik zou graag meer controles willen hebben**

3. **Heeft u een infuus gekregen?**
   - **Ja**
   - **Nee**

4. **Vond u het vervelend een infuus te krijgen?**
   - **Helemaal niet**
   - **Een beetje**
   - **Nogal**
   - **Heel erg**

5. **Zou u liever een MRI scan zonder contrastmiddel hebben, indien het veilig is?**
   - **Ja**
   - **Nee**

6. **Wat vond u van de wachttijd tussen het plaatsen van de infuus en het maken van de MRI?**
   - **Erg lang**
   - **Lang**
   - **Niet lang, niet kort**
   - **Kort**
   - **Erg kort**

7. **Had u klachten tijdens of direct na de MRI scan (bijv. misselijkheid)?**
   - **Helemaal niet**
   - **Een beetje**
   - **Nogal**
   - **Heel erg**

8. **Wat was uw ervaring met de MRI scan zelf?**
   - **Helemaal niet vervelend**
   - **Een beetje vervelend**
   - **Nogal vervelend**
   - **Heel erg vervelend**

9. Wat vond u van de duur van de MRI scan (het liggen in de scanner zelf)?
- **Erg lang**
- **Lang**
- **Niet lang, niet kort**
- **Kort**
- **Erg kort**

10. Indien u stress heeft door de MRI, waarom is dat zo?
- **Angst voor uitslag/slecht nieuws**
- **Reistijden naar het MRI-centrum**
- **Angst voor het scannen/de machine zelf/geluiden**
- **Angst voor de kleine ruimte/claustrofobie**
- **Angst voor infuusprikken**

11. Weet u van mogelijke nadelen voor patiënten door gadolinium contrastmiddel?
- □ **Ja**
- □ **Nee**

12. Hier is plek voor uw eigen commentaren:

____

____

____

____
