# Supplementary material for: The patients’ experience of neuroimaging of primary brain tumors: a cross-sectional survey study
Source: J Neurooncol. 2023 Mar 28;162(2):307–15. doi: 10.1007/s11060-023-04290-x (PMC10167184; doi:10.1007/s11060-023-04290-x)
Supplement: Supplementary file 2 — Supplementary file2 (PDF 153 KB) [file 11060_2023_4290_MOESM2_ESM.pdf]

Supplementary table 1. Tumor entities with the number of patients and latest confirmed WHO –based diagnosis/tumor grade.

Tumor entity	Grade	Number of patients
Glioblastoma	4	18
Midline glioma, H3 K27-M mutant	4	1
Oligodendroglioma	3	13
Astrocytoma	3	7
Ependymoma, RELA fusion-positive	3	1
Pineal Parenchymal Tumor of Intermediate Differentiation	3	1
Diffuse astrocytoma	2	20
Oligodendroglioma	2	17
pleomorphic xanthoastrocytoma	2	3
Pineocytoma	2	1
Ganglioglioma	1	4
Pilocytic astrocytoma	1	3
Dysembryoplastic neuroepithelial tumor	1	2
Gemistocytic astrocytoma	1	1
Astrocytoma	1	1
Unknown (radiological low-grade gliomas)	-	7
