# Supplementary material for: The patients’ experience of neuroimaging of primary brain tumors: a cross-sectional survey study
Source: J Neurooncol. 2023 Mar 28;162(2):307–15. doi: 10.1007/s11060-023-04290-x (PMC10167184; doi:10.1007/s11060-023-04290-x)
Supplement: Supplementary file 3 — Supplementary file3 (PDF 134 KB) [file 11060_2023_4290_MOESM3_ESM.pdf]

Supplementary table 2. Statistical tests between four different groups: Male vs female, HGG vs LGG, having had less than 10 scans vs more than 30 scans, and being younger than 25 years vs being older than 55 years.

			P-values			
**Question number**	**Question**	**Test**	**Male vs female** (n=100; 61 vs 39)	**HGG vs LGG** (n=100; 41 VS 59)	**≤10 scans vs ≥30 scans** (n=43; 33 VS 10)	**≤35 years or ≥55 years** (n=58; 32 VS 26)
2	Would you prefer to go to the hospital for MRI checkups less often or more often?	Chi-squared	0.285	0.342	0.046	0.945
4	Did you find it unpleasant to receive a cannula?	Mann-Whitney U test	0.003*	0.479	0.854	0.744
5	Would you prefer an MRI scan without a contrast agent, if diagnostically non-inferior?	Chi-squared	0.302	0.943	0.14	0.559
6	How did you feel about the wait time between placing the cannula and taking the MRI?	Mann-Whitney U test	0.247	0.821	0.788	0.086

7	Do you believe to have had any symptoms during or immediately after the MRI scan (e.g., nausea)?	Mann-Whitney U test	0.007*	0.103	0.724	0.703
8	What was your experience with the MRI scan itself?	Mann-Whitney U test	0.022	0.895	0.229	0.091
9	How did you feel about the duration of the MRI scan (lying in the scanner itself)?	Mann-Whitney U test	0.099	0.295	0.505	0.059
10.1	Did you fear the outcome/bad news?	Chi-squared	<0.001*	0.28	0.148	0.82
10.2	Did you experience stress due to the travel times to the MRI center?	Chi-squared	0.032	0.396	1	0.582
10.3	Did you experience fear of the scan/machine itself/noises?	Chi-squared	0.306	0.733	0.32	0.163

10.4	Did you fear the small space/experience claustrophobia?	Chi-squared	0.103	0.025	0.32	0.367
10.5	Did you fear getting a cannula ?	Chi-squared	0.103	1	0.04	0.681
10.6	Patients did not experience any stress during the MRI	Chi-squared	0.003*	0.481	0.481	0.475
11	Do you know of any possible adverse effects on patients from gadolinium contrast agents?	Chi-squared	1	0.593 [a] (27 VS 31)	not computable [b]	1 [a] (20 VS 14)

[a] This question was added later and only filled in by 58 patients. In brackets the distribution was denoted. [b] All 44 patients answered no. The Chi-squared test could therefore not be computed

* Significance level was set at 0.01.

HGG = high-grade glioma; LGG = low-grade glioma; MRI = magnetic resonance imaging; IV = intravenous
